# Cell-Specific Extracellular Vesicles Targeting Strategies for Immune Modulation in Inflammatory Diseases

**DOI:** 10.3390/pharmaceutics18060697

**Published:** 2026-06-05

**Authors:** Junha Lee, Suan Kwon, Yoosoo Yang, Jiwoong Choi

**Affiliations:** 1 Department of MetaBioHealth, School of Medicine, Sungkyunkwan University, Suwon 16419, Republic of Korea; doa04095@g.skku.edu; 2 School of Medicine, Kyungpook National University, Daegu 41944, Republic of Korea; suankwon@naver.com; 3 Department of Integrative Biotechnology, Sungkyunkwan University, Suwon 16419, Republic of Korea; 4 Department of Immunology, Cell & Matrix Research Institute, School of Medicine, Kyungpook National University, Daegu 41944, Republic of Korea

**Keywords:** extracellular vesicles, inflammatory diseases, surface engineering, cell targeting, chemical conjugation

## Abstract

Extracellular vesicles (EVs) have attracted considerable attention as natural nanocarriers for immune modulation owing to their intrinsic biocompatibility, nanoscale size, and capacity to transport diverse bioactive cargos. In inflammatory diseases, EV-based therapeutics provide unique opportunities to regulate dysregulated immune responses; however, their clinical translation remains constrained by limited cell-specific targeting efficiency and uncontrolled biodistribution. Achieving precise and selective delivery to immune cells and other inflammation-associated cellular components within diseased tissues is therefore critical for maximizing therapeutic efficacy while minimizing off-target effects. This review comprehensively summarizes recent advances in cell-specific EV-targeting strategies for immune modulation in inflammatory diseases, with a particular focus on active targeting approaches enabled by EV surface engineering. A range of targeting ligands, including antibodies, peptides, aptamers, glycans, and membrane proteins, is discussed in the context of enhancing selective interactions between EVs and specific immune cell subsets. Special emphasis is placed on cell-directed targeting strategies toward diverse immune cell populations, including macrophages and T cells, highlighting how rational control of EV–cell interactions can be utilized to reprogram immune phenotypes, suppress pathological inflammation, and restore immune homeostasis. Accordingly, this review integrates recent progress in cell-specific EV targeting into a coherent conceptual framework, which may assist researchers in the rational design of EV-based immunomodulatory therapeutics.

## 1. Introduction

Inflammatory diseases are characterized by dysregulated immune responses that lead to persistent tissue damage and impaired resolution of inflammation [1]. These conditions, including autoimmune disorders, metabolic inflammation, and chronic inflammatory syndromes, arise from complex interactions among diverse immune cell populations, such as macrophages, dendritic cells, and T lymphocytes [1,2,3]. Prolonged activation of these immune networks establishes a highly polarized microenvironment enriched with proinflammatory mediators, ultimately driving irreversible tissue remodeling and organ dysfunction. Despite significant advances in immunomodulatory therapies, current treatment strategies often rely on systemic administration of anti-inflammatory or immunosuppressive agents, which lack cell specificity and are frequently associated with off-target effects and limited therapeutic efficacy [4,5,6]. Moreover, the broad suppression of systemic immunity inherently increases patient susceptibility to severe opportunistic infections, underscoring an urgent clinical need for precise, cell-directed therapeutic platforms.

To overcome these critical translational limitations, extracellular vesicles (EVs) have attracted considerable attention as natural nanocarriers for immune modulation [7,8,9]. EVs are lipid bilayer-enclosed nanoparticles released through tightly regulated cellular processes, including endosomal sorting and membrane budding, and function as fundamental mediators of intercellular communication [10]. Because EVs comprise heterogeneous vesicle populations with distinct cellular origins and biogenetic routes, including endosome-derived exosomes and plasma membrane-derived microvesicles, the term “EVs” is used broadly in this review to refer to these diverse vesicles. EVs encapsulate and transfer a complex repertoire of biomolecules, including proteins, lipids, mRNAs, miRNAs, and other regulatory molecules, thereby protecting these cargos from enzymatic degradation and enabling functional communication with recipient cells. Mechanistically, EVs can contribute to immune balance by modulating NF-κB- and MAPK-associated inflammatory signaling, metabolic reprogramming, and gene expression profiles in target cells [11,12]. These EV-mediated signals can suppress excessive cytokine production, promote inflammation-resolving macrophage phenotypes, regulate antigen-presenting cell activation, restrain pathogenic effector T cell responses, and support regulatory T cell functions, thereby shifting dysregulated immune networks toward resolution and tissue homeostasis [13,14,15]. The cellular source of EVs, therefore, represents a key determinant of their therapeutic behavior, as mesenchymal stromal-cell-derived EVs are frequently associated with tissue repair and immunoregulation, immune-cell-derived EVs can retain cell type-specific immunological signals, epithelial or tissue-resident-cell-derived EVs may reflect local inflammatory environments, and engineered producer-cell-derived EVs provide modular platforms for controlled cargo loading and surface functionalization. These source-dependent biological signatures and functional biomolecules can be harnessed to suppress excessive inflammation, promote immune tolerance, and restore tissue homeostasis in inflammatory diseases.

Furthermore, beyond their natural cargo, EVs can be actively engineered as versatile drug delivery vehicles by loading exogenous therapeutic agents into their aqueous core or incorporating lipophilic drugs into their lipid bilayer membrane [16,17,18]. Various loading strategies, including electroporation, sonication, membrane permeabilization, and donor cell engineering, have been developed to enhance cargo encapsulation efficiency and functional delivery [19].

Delivery systems, including improved stability, prolonged circulation, and reduced clearance by the mononuclear phagocyte system [20,21]. Nevertheless, when administered systemically, EVs often exhibit predominantly passive biodistribution and tend to accumulate in off-target organs such as the liver, spleen, and lungs, largely due to nonspecific uptake by resident phagocytic cells [22,23]. This non-selective distribution significantly limits their effective delivery to disease-relevant immune cell populations. Consequently, achieving precise and selective delivery to specific immune cell subsets and other inflammation-associated cellular components within the pathological microenvironment has emerged as a central requirement for maximizing therapeutic efficacy while minimizing off-target effects and systemic toxicity.

**Figure 1:**
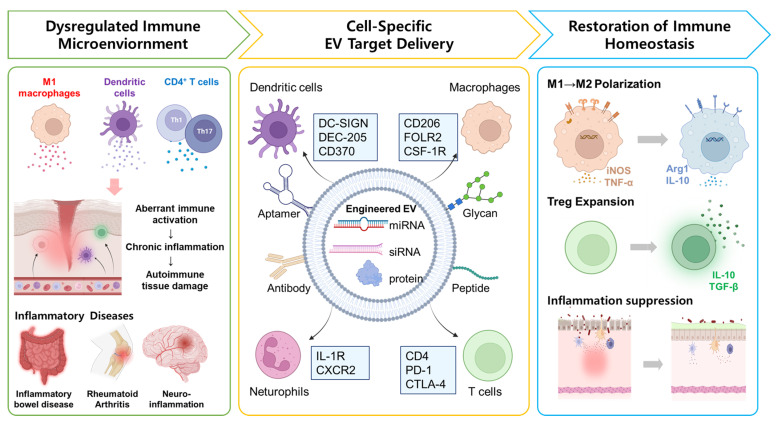
Cell-specific delivery of engineered EVs for the restoration of tissue homeostasis (created in BioRender. Choi, J. (2026) https://BioRender.com/5q8su3h (accessed on 11 May 2026).

Achieving this required precision necessitates active targeting approaches enabled by EV surface engineering. By strategically decorating the EV membrane with specific recognition motifs, these nanocarriers can transition from passive delivery vehicles into highly selective immunomodulatory platforms. To accomplish this, various engineering strategies have been developed to functionalize the EV surface, ranging from genetic modification of parent cells to post-isolation techniques such as chemical conjugation utilizing antibodies, peptides, and glycans, to physical methods like membrane fusion. These rational modifications are designed to exploit specific receptor and ligand interactions, thereby significantly enhancing selective binding between EVs and target immune cell subsets. By directing customized immunomodulatory cargos precisely toward critical myeloid populations, such as macrophages and dendritic cells, as well as lymphoid populations, including T cell subsets and natural killer cells, engineered EVs can efficiently reprogram immune phenotypes, suppress pathological inflammation, and restore immune homeostasis (Figure 1).

This review comprehensively summarizes recent advances in cell-specific EV-targeting strategies for inflammatory diseases. We provide a detailed overview of current surface engineering techniques and their direct mechanisms in facilitating selective EV and cell interactions. Furthermore, we explore the emerging therapeutic applications of these engineered nanocarriers across a spectrum of inflammatory disorders, highlighting their potential in inflammatory bowel disease and rheumatoid arthritis. Although diverse immune and stromal cell populations, including B cells, NK cells, regulatory T cells, and fibroblast-like synoviocytes, contribute to inflammatory disease progression, this review places particular emphasis on macrophage-targeting strategies because macrophages are highly plastic central regulators of inflammatory microenvironments and currently represent the most extensively investigated immune targets in engineered EV-based immunomodulatory therapies. By integrating these targeted approaches into a coherent conceptual framework, this review aims to assist researchers in the rational design and clinical advancement of next-generation EV therapeutics, concluding with a discussion on current challenges and future perspectives in the field.

## 2. Engineering Strategies for Cell-Specific EV Delivery

**Figure 2:**
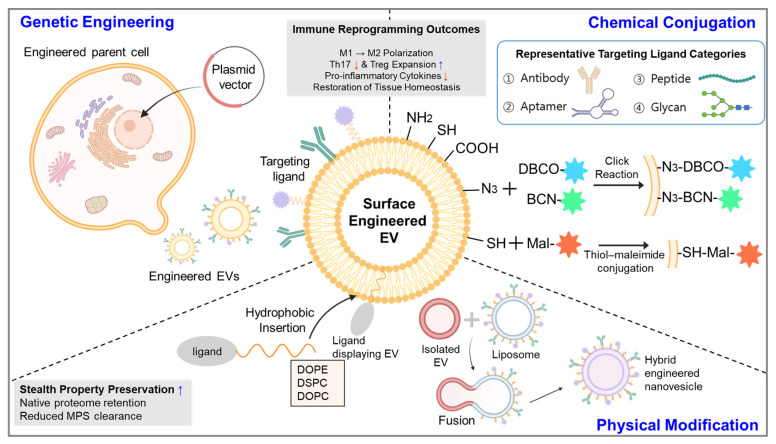
Surface engineering strategies for cell-targeted EV delivery (created in BioRender. Choi, J. (2026) https://BioRender.com/68f8igp (accessed on 11 May 2026)).

Achieving precise tissue- and cell-targeting is the most critical prerequisite for the successful clinical application of EV-based therapeutics. EVs can be obtained from diverse biological or biofluid sources, and the choice of isolation method directly influences their purity, yield, structural integrity, and suitability for subsequent engineering [24,25]. Differential ultracentrifugation and density-gradient separation are widely used for EV enrichment, but they can be time-consuming and may affect vesicle recovery depending on processing conditions. In contrast, size-exclusion chromatography and ultrafiltration provide relatively gentle separation approaches that better preserve vesicle integrity, whereas polymer-based precipitation offers high recovery but may introduce protein or polymer contaminants. Affinity-based capture enables marker-selective EV isolation, although its scalability and potential bias toward specific EV subpopulations should be carefully considered. Native EVs can exhibit intrinsic tropism through endogenous surface molecules, parent cell-derived biological signatures, and cargo-mediated signaling functions. However, these interactions are often context-dependent, heterogeneous, and insufficiently controllable for reproducible therapeutic delivery to defined immune cell subsets. Thus, the purpose of EV surface engineering is not to create cell selectivity de novo, but to enhance, redirect, and standardize the intrinsic EV–cell communication properties for more predictable therapeutic targeting. In addition, distinct EV subtypes, including exosomes, microvesicles, and apoptotic bodies, differ in their biogenesis, membrane composition, and intracellular trafficking properties, all of which may influence engineering efficiency and targeting behavior, despite the broad use of the term “EV” throughout this review in accordance with common field terminology [26,27,28]. These engineering approaches are broadly categorized into three main methodologies. The first is the genetic engineering of parent cells prior to EV isolation. The subsequent methods involve post-isolation modifications, specifically chemical conjugation and physical membrane modifications. Each strategy utilizes distinct mechanisms to anchor specific recognition ligands to the EV surface, offering unique advantages and specific technical challenges depending on the nature of the targeting moiety and the required clinical application (Figure 2).

### 2.1. Genetic Engineering

Genetic engineering represents a highly effective pre-isolation strategy that exploits the endogenous biosynthetic and vesicular trafficking pathways of parent cells to display targeting ligands directly on the membrane of secreted EVs. This approach is typically achieved by constructing plasmid or viral vectors encoding fusion proteins in which a targeting moiety, such as a homing peptide or antibody fragment, is genetically fused to well-characterized EV-associated membrane proteins [29,30,31]. Upon expression in engineered donor cells, these fusion proteins are trafficked through the endosomal system and incorporated into intraluminal vesicles during multivesicular body formation, ultimately enabling their stable presentation on the outer lipid bilayer of secreted EVs. Because ligand incorporation occurs during natural EV biogenesis, this biologically integrated process ensures relatively uniform ligand distribution, correct topological orientation, and high membrane stability without requiring post-isolation chemical modifications that may compromise EV integrity or function.

Importantly, this strategy enables rational molecular-level design, allowing the integration of multiple functional domains within a single construct and facilitating the generation of EVs with enhanced targeting capability and therapeutic potential. Representative scaffold proteins such as Lamp2b, PDGFR, CD63, and CD9 are commonly employed due to their intrinsic trafficking routes and membrane topology within the endosomal system [32,33,34,35]. Following genetic engineering, these proteins are synthesized in the endoplasmic reticulum and subsequently trafficked through the Golgi apparatus, where they undergo proper folding and post-translational modifications. They are then directed to early endosomes and sorted into intraluminal vesicles during multivesicular body formation, a process mediated by ESCRT-dependent or -independent mechanisms. Crucially, the membrane topology of these scaffold proteins ensures that their luminal or extracellular domains are oriented outward upon EV secretion, thereby enabling the functional display of genetically fused targeting ligands on the EV surface. Lamp2b, in particular, has been extensively utilized as a surface display scaffold, as its N-terminal domain is naturally exposed on the EV exterior, allowing the fusion of targeting peptides or antibody fragments for selective cell or tissue recognition [36]. Accordingly, Lamp2b-based fusion strategies should be interpreted primarily in the context of endosome-derived exosomes, as ligand display through this scaffold depends on trafficking through the endosomal and multivesicular body pathway and may not be directly applicable to microvesicles generated by plasma membrane budding or apoptotic bodies. The transmembrane domain of PDGFR represents an alternative anchoring strategy that enables stable membrane insertion of targeting ligands, on the EV membrane, with studies demonstrating efficient surface presentation of peptides such as GE11 for EGFR-specific targeting [37,38]. Tetraspanins such as CD63 and CD9 further expand this platform, as they are highly enriched in EV membranes and actively participate in vesicle biogenesis and cargo sorting [33]. Engineering these proteins allows not only surface functionalization but also the incorporation of therapeutic cargos, including nucleic acids and gene-editing components. Beyond their role as structural scaffolds, these proteins contribute to the stabilization and organization of membrane microdomains, thereby enhancing the functional display and biological performance of engineered EVs.

Despite these advantages, genetic engineering-based EV surface functionalization is subject to several inherent biological and translational limitations. A primary constraint is that this strategy is largely restricted to genetically encodable ligands, thereby limiting its applicability to peptide- and protein-based targeting moieties while excluding non-protein ligands such as glycans or synthetic small molecules. In addition, the overexpression of fusion proteins can impose metabolic stress on donor cells, potentially leading to reduced cell viability, impaired EV secretion, and disruption of endogenous vesicle sorting pathways, which may ultimately alter EV cargo composition or reduce overall yield. A particularly critical challenge arises from intracellular processing, where targeting peptides fused to scaffolds are exposed to proteolytic degradation within endosomal and lysosomal compartments during EV biogenesis, resulting in diminished ligand stability and targeting efficiency. To address this issue, stabilizing strategies such as the incorporation of glycosylation motifs (e.g., GNSTM sequence) at the N-terminus of fusion constructs have been employed to protect targeting peptides from proteolytic cleavage and enhance surface display stability [39]. Nevertheless, these additional modifications inevitably increase vector design complexity and may further influence protein folding and intracellular trafficking. Collectively, while genetic engineering provides a biologically integrated and structurally robust approach for EV surface functionalization, careful optimization of scaffold selection, fusion design, and intracellular processing is essential to balance targeting efficiency with cellular compatibility and translational feasibility.

### 2.2. Chemical Conjugation

While genetic engineering modifies the parent cells, chemical conjugation involves the direct functionalization of isolated EVs. This post-isolation strategy provides broad modularity, allowing for the attachment of a diverse array of targeting ligands, which cannot be achieved through genetic modification alone [40]. In practice, chemical conjugation exploits reactive groups naturally present on EV membrane proteins and lipids, including primary amines, carboxyl groups, and sulfhydryl groups, as well as bioorthogonal handles introduced by metabolic labeling or lipid insertion. The most prevalent chemical approaches utilize click chemistry, such as strain-promoted azide-alkyne cycloaddition (SPAAC), and crosslinking reactions targeting primary amines or sulfhydryl groups on the EV surface proteins [41,42]. For example, EDC/NHS coupling forms amide bonds between carboxyl and amine groups, thiol–maleimide chemistry enables conjugation to sulfhydryl groups, and SPAAC allows copper-free ligation between azide- and cyclooctyne-modified components under mild aqueous conditions. These methods can be used to attach antibodies, peptides, aptamers, glycans, imaging probes, or therapeutic molecules to EV surfaces. Although chemical conjugation offers high coupling efficiency and rapid functionalization, the reaction conditions must be carefully optimized. Excessive chemical modification or the use of harsh solvents can potentially alter the structural integrity of the lipid bilayer, induce EV aggregation, or mask the biological activity of intrinsic surface molecules.

#### 2.2.1. Antibody

Antibodies remain one of the most widely utilized targeting ligands for EV surface functionalization due to their unparalleled specificity and high binding affinity toward defined cellular receptors [43,44]. By conjugating monoclonal antibodies onto the EV membrane, these nanocarriers can be selectively directed to specific cell populations, including distinct immune subsets and diseased tissues. Conventionally, antibody immobilization has been achieved through non-specific chemical crosslinking approaches, such as carbodiimide-mediated (EDC/NHS) coupling, which forms covalent bonds between carboxyl groups on antibodies and primary amines on EV surface proteins [45]. While this strategy is straightforward and broadly applicable, it often results in random antibody orientation and heterogeneous surface distribution, which can significantly compromise antigen-binding efficiency due to steric hindrance or epitope masking.

To overcome these limitations, recent efforts have focused on developing site-specific conjugation strategies that enable controlled antibody orientation. A commonly employed approach involves the selective reduction in interchain disulfide bonds within the antibody hinge region to generate free thiol groups, which can subsequently react with maleimide-functionalized EV surfaces [46,47]. This thiol–maleimide coupling ensures directional immobilization of antibodies through the Fc region, thereby preserving the outward-facing orientation of antigen-binding domains and maximizing target recognition. Despite these advancements, the use of full-length antibodies presents intrinsic biological challenges. The Fc domain can interact with Fc receptors expressed on monocytes, macrophages, and other components of the mononuclear phagocyte system, leading to rapid clearance and off-target uptake [48,49]. To mitigate these issues, smaller antibody derivatives, including single-chain variable fragments (scFvs), Fab fragments, and nanobodies, have been increasingly adopted [50,51,52]. These engineered formats retain high target specificity while reducing molecular size, immunogenicity, and Fc-mediated non-specific interactions. Furthermore, their compact structure improves tissue penetration and facilitates higher ligand density on the EV surface.

Collectively, antibody-based EV engineering offers a highly versatile and clinically relevant targeting strategy, combining the well-established therapeutic efficacy of antibodies with the intrinsic delivery capabilities of EVs. However, achieving optimal functional performance requires careful consideration of conjugation chemistry, ligand orientation, and immunological interactions, as these factors critically influence targeting specificity, pharmacokinetics, and in vivo therapeutic outcomes.

#### 2.2.2. Peptide

Peptides have emerged as one of the most versatile targeting ligands for EV surface engineering due to their small molecular size, high binding specificity, and ease of synthesis and modification [53,54,55]. Compared to full-length antibodies, peptides exhibit significantly reduced steric hindrance, enabling higher surface density and improved accessibility to target receptors. In addition, their low immunogenicity and minimal Fc-mediated interactions contribute to reduced off-target uptake and prolonged circulation. Importantly, peptide sequences can be rationally designed based on natural ligand–receptor interactions or identified through high-throughput screening techniques such as phage display, allowing precise targeting toward specific cell types or pathological microenvironments. These characteristics make peptides particularly attractive for applications requiring efficient tissue penetration and adaptable targeting performance. To translate these advantages into effective EV targeting systems, post-isolation engineering strategies have been widely explored to enable stable and efficient peptide display on EV surfaces [56]. Typically, EV surfaces are first activated through carbodiimide chemistry to introduce reactive functional groups, followed by modification of peptides with complementary moieties such as azide or alkyne groups [57]. Subsequent azide–alkyne cycloaddition reactions, including SPAAC or copper-catalyzed azide–alkyne cycloaddition, enable the formation of stable covalent linkages between peptides and EV membranes, allowing efficient and controlled surface functionalization. In addition to click chemistry, alternative conjugation strategies, including thiol–maleimide coupling and affinity-based anchoring, have also been explored to achieve stable peptide immobilization without compromising EV integrity.

However, peptides remain susceptible to enzymatic degradation, may exhibit relatively lower binding affinity compared to full-length antibodies, and can undergo conformational instability in complex biological environments. Therefore, the development of environment-responsive or stimuli-activatable peptide designs, such as protease-cleavable, pH-sensitive, or redox-responsive systems, is required to enhance targeting specificity and stability within pathological microenvironments.

#### 2.2.3. Aptamers

Aptamers, often referred to as chemical antibodies, are short single-stranded DNA or RNA oligonucleotides, typically 20–100 nucleotides in length, that fold into well-defined three-dimensional structures such as stem–loops, G-quadruplexes, or pseudoknots [58,59,60]. These secondary and tertiary structures enable aptamers to recognize and bind target molecules, including proteins, lipids, and even whole cells, with high affinity and specificity through a combination of hydrogen bonding, electrostatic interactions, and shape complementarity. Aptamers are most commonly identified through the in vitro selection process known as Systematic Evolution of Ligands by EXponential enrichment, in which large combinatorial oligonucleotide libraries undergo iterative cycles of target binding, partitioning, amplification, and enrichment to isolate high-affinity binders [61]. This entirely synthetic selection process enables precise control over binding characteristics and eliminates the need for biological immunization, distinguishing aptamers from conventional antibodies.

In the context of EV engineering, aptamers provide a chemically defined and highly tunable platform for surface functionalization. Unlike protein-based ligands, aptamers can be readily synthesized with site-specific chemical modifications at the 5′ or 3′ termini, or within the sugar–phosphate backbone, allowing precise control over conjugation and orientation [62,63]. These modifications enable efficient coupling to EV membranes through established chemical conjugation strategies. For example, aptamers functionalized with terminal amine groups can be covalently linked to carboxyl groups present on EV surface proteins or lipids via carbodiimide chemistry, such as EDC/NHS-mediated amide bond formation [64]. Alternatively, azide- or alkyne-modified aptamers can be conjugated using bioorthogonal click chemistry, including copper-free SPAAC, enabling highly efficient and selective surface modification under mild conditions that preserve EV integrity. These diverse chemistries allow not only stable attachment but also precise control over ligand density and spatial orientation, which are critical parameters influencing targeting efficiency and cellular uptake.

Despite these advantages, aptamers are inherently susceptible to nuclease-mediated degradation and may exhibit limited stability in complex biological environments. To address these challenges, chemical modifications such as 2′-fluoro, 2′-O-methyl, or phosphorothioate backbone substitutions have been introduced to enhance nuclease resistance [63]. Additional strategies, including PEGylation or incorporation into protective nanostructures, can further improve circulation stability and functional performance. Collectively, while aptamers offer a versatile and chemically precise platform for EV surface engineering, optimizing their stability and conjugation strategy remains essential for achieving robust in vivo efficacy and translational applicability.

#### 2.2.4. Glycans

The innate immune system relies heavily on carbohydrate recognition, a dynamic process governed by various pattern recognition receptors and C-type lectins predominantly expressed on myeloid cells [65,66]. Therefore, functionalizing the EV surface with specific glycans represents a biomimetic strategy for targeting myeloid-derived antigen-presenting cells (APCs), particularly those expressing carbohydrate-binding receptors [67,68,69]. To achieve controlled glycosylation of EV surfaces, several chemical and biologically assisted engineering strategies have been developed. One conventional approach involves direct chemical conjugation of glycan moieties onto nanocarrier surfaces through well-established covalent linkage strategies. Monosaccharides possess multiple reactive functional groups, particularly hydroxyl and amino groups, which enable stable conjugation with carrier systems via diverse chemical reactions. For instance, glycan ligands can be introduced through direct amide bond formation using activated ester or carboxyl derivatives, thiourea linkage formation via isothiocyanate-functionalized saccharides, or amidization reactions between amino-functionalized sugars and carboxyl-bearing nanocarriers. However, although monosaccharide conjugation provides a chemically accessible route for EV surface modification, its targeting specificity should be interpreted with caution because glycan recognition often requires discrimination among highly diverse carbohydrate structures defined by monosaccharide sequence, branching, and glycosidic linkage patterns rather than by a single terminal sugar [70]. In addition, endogenous galactose-terminated glycans present in the cellular glycocalyx and soluble glycoproteins may compete with galactose-decorated EVs, further limiting their target selectivity under physiological conditions. Thus, simple monosaccharide decoration should be regarded as a preliminary glycomimetic strategy, and further control over glycan structure and surface presentation will be required to achieve more predictable EV targeting.

Complementary to these direct modification strategies, metabolic glycoengineering has emerged as a powerful approach for introducing bioorthogonal functional groups onto EV surfaces in a controlled and biologically integrated manner [71,72,73]. In this strategy, parent cells are cultured in the presence of unnatural monosaccharide precursors, such as peracetylated N-azidoacetylmannosamine (Ac_4_ManNAz), which enter endogenous glycan biosynthetic pathways and are metabolically converted into sialic acid analogs bearing bioorthogonal functional groups. These modified sialic acids are then naturally incorporated into cell-surface glycoconjugates and subsequently displayed on the membrane of secreted EVs. This process enables the stable and uniform presentation of reactive chemical handles, such as azide groups, on the EV surface without disrupting membrane proteins or lipid organization. The introduced bioorthogonal groups serve as highly selective anchoring sites for subsequent functionalization via click chemistry, most commonly through SPAAC or copper-catalyzed azide–alkyne cycloaddition. These reactions enable rapid, efficient, and site-specific conjugation of targeting ligands, imaging probes, or therapeutic molecules under mild conditions, thereby preserving the structural and biological integrity of EVs. By decoupling functional group installation from ligand conjugation, this strategy provides a modular and scalable platform that allows precise control over surface functionalization. Such an approach enables in situ labeling and facilitates the integration of diverse functionalities, making it highly adaptable for advanced EV-based drug delivery and diagnostic applications.

### 2.3. Physical Membrane Modification

While chemical conjugation offers robust covalent linkages, it poses an inherent risk of denaturing sensitive EV surface proteins and compromising their native biological activities. To preserve the intrinsic protein landscape and structural integrity of the EV membrane, physical and non-covalent engineering strategies have emerged as compelling alternatives. Within this framework, lipid post-insertion stands out as a primary strategy for functionalizing vesicles without compromising their biological fidelity [74,75]. In this method, targeting ligands such as antibodies, peptides, or aptamers are covalently tethered to the hydrophilic terminus of a bifunctional polymer spacer, typically polyethylene glycol (PEG), which is anchored to a hydrophobic phospholipid moiety. The most prevalent anchor, 1,2-distearoyl-sn-glycero-3-phosphoethanolamine (DSPE), is particularly well-suited for this application due to its long, saturated C18 acyl chains, which promote stable membrane insertion and relatively prolonged retention within lipid bilayers [76,77]. This amphiphilic architecture enables efficient membrane integration, as the hydrophobic tails spontaneously partition into the EV lipid bilayer upon co-incubation, driven by hydrophobic interactions. This process allows for stable, preferential outward display of ligands without requiring direct chemical reactions on the EV surface, thereby preserving the native proteome and membrane functionality. Furthermore, the integrated PEG layer provides steric stabilization, minimizes opsonization or nonspecific protein adsorption, and effectively reduces premature clearance by the mononuclear phagocyte system.

In parallel, membrane fusion-based strategies have emerged as a powerful approach to engineer EV surfaces by physically integrating them with synthetic lipid vesicles [78,79,80,81]. This process is typically achieved through physicochemical methods such as extrusion, sonication, or repeated freeze–thaw cycles, which induce transient membrane destabilization followed by lipid mixing and bilayer reconstitution. The resulting hybrid vesicles exhibit intermixed lipid components derived from both EVs and liposomes, while maintaining key membrane proteins from the parent EVs. Importantly, such hybridization allows modulation of EV surface properties that are otherwise difficult to control through genetic engineering alone. For example, incorporation of exogenous lipids with defined physicochemical characteristics can alter membrane charge, fluidity, and surface functionality, thereby influencing cellular uptake and interactions with recipient cells. For instance, incorporation of cationic or ionizable lipids can enhance electrostatic interactions with negatively charged cell membranes, thereby promoting cellular uptake [81]. Similarly, increasing the proportion of unsaturated phospholipids can improve membrane fluidity, facilitating membrane fusion and endosomal escape following internalization [27]. Beyond conventional aliphatic lipids like DSPE, sterol derivatives serve as paramount structural modulators that rigorously regulate the physicochemical and thermodynamic properties of phospholipid bilayer systems [82]. Mechanistically, the incorporation of specific sterols dictates bilayer ordering, membrane thickness, and packing density, which are essential for engineering stable vesicle platforms with tailored fluidity and reduced permeability. Building upon these biophysical principles, recent biophysical evaluations have explicitly demonstrated that substituting conventional lipids with specific sterol-modified derivatives significantly improves the overall structural stability and elasticity of lipid-based vesicles [83]. Crucially, the utilization of these sterol derivatives translates into concrete therapeutic and translational achievements by drastically enhancing in vivo stability and targeted delivery efficiency [84]. Taken together, these findings highlight that precise tuning of lipid composition through membrane fusion and sterol-driven hydrophobic insertion enables programmable control over EV uptake pathways, intracellular trafficking, and in vivo distribution, providing a mechanistic basis for engineering EV functionality beyond native biological constraints.

Building on these mechanistic insights, hydrophobic insertion and membrane fusion strategies can be viewed as complementary non-covalent approaches for EV surface engineering. Whereas lipid post-insertion enables the controlled and stable display of targeting ligands with minimal perturbation of membrane proteins, membrane fusion provides a versatile means to reprogram the overall physicochemical properties of EV membranes through lipid compositional tuning. Together, these strategies offer a scalable and modular platform for functionalizing EVs while preserving their structural integrity and biological activity.

Beyond extracellular targeting, surface engineering can also influence the intracellular fate of EVs after cellular uptake. Depending on the engaged receptor, recipient cell type, and EV membrane properties, engineered EVs may enter cells through clathrin-mediated endocytosis, caveolae-dependent uptake, macropinocytosis, phagocytosis, lipid raft-mediated internalization, or direct membrane fusion [85,86]. Genetic engineering can promote receptor-dependent uptake by displaying defined ligands or peptides on the EV surface, whereas physical membrane modification can alter membrane charge, fluidity, and lipid composition, thereby affecting endosomal sorting, membrane fusion, and cargo release. Because a substantial fraction of internalized EVs may traffic to endolysosomal compartments, future EV designs should incorporate strategies that enhance endosomal escape or reduce lysosomal degradation, such as fusogenic lipids, pH-responsive components, membrane-destabilizing peptides, or organelle-targeting motifs, to improve the intracellular bioavailability of therapeutic cargos.

From a translational and industrial perspective, each EV surface engineering strategy presents a distinct set of biophysical advantages and manufacturing trade-offs that dictate its suitability for clinical applications [18]. Genetic engineering enables inherently stable, uniform, and topologically correct ligand display during vesicle biogenesis without the need for post-isolation processing. However, its translational implementation is often bottlenecked by the high cost of donor-cell engineering, variable or transient transgene expression levels, and rigorous regulatory requirements associated with GMOs and complex cell-banking systems. Conversely, chemical conjugation offers unparalleled modularity and robust covalent attachment of a diverse array of synthetic or biological ligands post-isolation. Nonetheless, achieving precise control over ligand density and spatial orientation remains highly challenging due to the intrinsic heterogeneity of reactive functional groups on the native EV membrane; furthermore, the potential toxicity of residual chemical reagents or excessive surface modification poses risks of compromising membrane integrity and altering pharmacokinetics. Within non-covalent frameworks, lipid post-insertion provides a highly scalable, mild, and cost-effective approach that preserves the native proteomic landscape of EVs with minimal immunogenicity risks. Yet, this strategy requires rigorous validation regarding long-term ligand retention and the potential shedding or dissociation of lipid anchors in complex physiological environments. Therefore, the optimal surface engineering strategy cannot be standardized; rather, it must be strategically selected by balancing the specific therapeutic objective, target cellular lineage, required ligand stability, acceptable manufacturing complexity, and regulatory feasibility.

## 3. Cell-Specific Targeting Strategies

Optimizing the efficacy of EV-based immunomodulatory therapeutics necessitates refining their intrinsically non-specific biodistribution profiles. Active targeting strategies, facilitated by functionalizing the vesicular surface with specific ligands, enable the selective recognition of target receptors and subsequently promote receptor-mediated endocytosis (Table 1). This targeted interaction significantly enhances the intracellular accumulation of therapeutic cargos within defined immune cell subsets, thereby improving therapeutic efficacy while minimizing off-target effects. In this section, we outline the key receptor–ligand axes exploited for the selective targeting of myeloid and lymphoid cell populations (Table 2). Specifically, we highlight how engaging distinct surface markers enables precise payload delivery, allowing for the controlled modulation of specific immune cell functions to help restore immune balance. Thus, the following subsections are organized not merely to describe immune cell biology, but to connect disease-relevant cellular functions with targetable receptor–ligand axes that can be exploited for EV surface engineering. In this context, EV–cell interactions should be evaluated not only by cellular uptake or biodistribution, but also by functional outcomes in recipient cells, including changes in cytokine secretion, receptor signaling, polarization state, antigen-presenting capacity, and T cell activation or suppression. Therefore, evidence linking engineered EV targeting to functional immune-cell modulation is essential for validating their therapeutic relevance.

### 3.1. Macrophages

Macrophages are highly plastic innate immune cells derived from circulating monocytes that serve as a critical first line of defense against invading pathogens and play essential roles in maintaining immune homeostasis [87]. These phagocytes detect pathogen- and damage-associated signals through a sophisticated array of pattern recognition receptors, thereby triggering downstream signaling cascades that orchestrate cytokine secretion, leukocyte recruitment, and the broader progression of the inflammatory response. Extending beyond host defense, macrophages are integral to resolving tissue injury by driving extracellular matrix remodeling and performing efferocytosis, which collectively re-establish structural integrity and immune homeostasis. A defining feature of macrophages is their remarkable phenotypic heterogeneity and functional plasticity, which are largely dictated by the surrounding microenvironment [88,89]. In response to distinct cytokine cues and pathological stimuli, macrophages undergo polarization into a spectrum of activation states, broadly categorized into classically activated pro-inflammatory M1 and alternatively activated anti-inflammatory M2 phenotypes. M1 macrophages are typically induced by microbial products or Th1 cytokines and are characterized by the production of pro-inflammatory mediators, thereby contributing to pathogen clearance and amplification of inflammatory responses. In contrast, M2 macrophages are driven by Th2 cytokines and are primarily involved in anti-inflammatory functions and tissue repair.

Under physiological conditions, a tightly regulated balance between these functional states is essential for maintaining immune homeostasis. However, sustained or excessive M1 activation is a major driver of chronic inflammation and tissue damage, contributing to the pathogenesis of various immune-related diseases such as autoimmune disorders, metabolic inflammation, and chronic inflammatory conditions [88]. This pro-inflammatory state is characterized by persistent production of cytokines such as TNF-α, IL-1β, and IL-6, along with reactive mediators, which collectively promote prolonged tissue injury and disruption of normal immune regulation. In contrast, M2 polarization plays a role in inflammation resolution and tissue repair through the secretion of anti-inflammatory cytokines such as IL-10 and TGF-β but is also associated with pathological tissue remodeling when dysregulated or persistently activated. This polarization-dependent surface phenotype provides a mechanistic link between macrophage biology and EV-targeting strategies. Because M1 and M2 macrophages display distinct, although partially overlapping, receptor profiles, engineered EVs can be designed to selectively engage activation-associated markers and deliver immunomodulatory cargos that suppress pro-inflammatory signaling, promote macrophage reprogramming, or modulate tissue-remodeling responses.

Building upon the functional heterogeneity of macrophages, selective targeting of specific macrophage subpopulations has emerged as a critical strategy for modulating immune responses. During pro-inflammatory polarization, M1 macrophages acquire a distinct surface proteome that provides highly specific docking sites for engineered nanocarriers to intercept the inflammatory cascade. Prominent among these molecular anchors are costimulatory molecules such as CD80 and CD86, which undergo robust upregulation and can be actively engaged using engineered affinity ligands to enhance targeting precision, thereby establishing a highly specific entry route for therapeutic intervention against excessive inflammation [90,91].

In contrast, alternatively activated (M2) macrophages are associated with immune suppression, tissue repair, and pathological remodeling, and thus represent key targets for therapeutic reprogramming. Among the most universally utilized markers for this purpose is the mannose receptor CD206, which exhibits high endocytic activity and can be efficiently targeted using mannose- or dextran-functionalized systems to facilitate cellular uptake [92,93,94]. In parallel, folate receptor-β serves as another highly specific docking site, as it is robustly expressed on M2 macrophages under inflammatory conditions, enabling folic acid-functionalized nanocarriers to achieve high-affinity engagement and subsequent receptor-mediated endocytosis, thereby facilitating the precise intracellular delivery of immunomodulatory therapeutics [95,96]. Similarly, CD163, a scavenger receptor responsible for the clearance of hemoglobin–haptoglobin complexes, is selectively upregulated on M2 macrophages and serves as a robust entry point when engaged with tailored affinity ligands [97,98]. In the realm of glycan recognition, the C-type lectin receptor CD301 provides another highly efficient targeting axis; exploiting this receptor with galactose-modified ligands drives robust nanocarrier internalization and maximizes the intracellular accumulation of therapeutics [99,100]. Expanding beyond clearance and recognition mechanisms, targeting cytokine signaling pathways offers a direct route for profound phenotypic reprogramming. Specifically, interleukin-4 receptor α (IL-4Rα) can be strategically engaged using engineered peptide ligands to not only facilitate highly specific cellular uptake but also competitively disrupt native immunosuppressive signaling, thereby actively reversing the M2 state [101]. Together, these receptor–ligand interactions highlight the versatility of macrophage-targeting strategies and provide a foundation for the development of precise and context-dependent immunomodulatory therapies.

### 3.2. Neutrophils

Neutrophils are the most abundant circulating innate immune cells and act as rapid first responders to infection and tissue injury [102,103]. Upon inflammatory stimulation, they are rapidly recruited from the bloodstream to affected tissues through chemokine gradients and adhesion cascades. This recruitment process is tightly orchestrated by chemokine–receptor interactions, particularly the CXCL8–CXCR1/2 axis, which guides neutrophil chemotaxis toward inflamed tissues [104,105]. Once localized, neutrophils exert their primary effector functions, including phagocytosis of pathogens, degranulation of antimicrobial proteins, and production of reactive oxygen species. In addition to these classical antimicrobial activities, neutrophils can release neutrophil extracellular traps (NETs), which consist of decondensed chromatin decorated with antimicrobial enzymes and serve to immobilize and eliminate invading microorganisms [103]. Beyond direct pathogen clearance, neutrophils also contribute to tissue repair and resolution processes by removing cellular debris and secreting mediators that promote angiogenesis and immune regulation. Furthermore, neutrophils actively engage in crosstalk with other immune cells, including macrophages, dendritic cells, and lymphocytes, thereby shaping both innate and adaptive immune responses.

However, under chronic inflammatory conditions, neutrophils shift from protective effectors to key drivers of disease pathology. Persistent recruitment and activation of neutrophils lead to excessive release of proteolytic enzymes, such as neutrophil elastase and myeloperoxidase, as well as sustained production of reactive oxygen species, which collectively contribute to tissue damage and inflammatory amplification [106,107]. This tissue injury, in turn, triggers a pathogenic cycle where continuous neutrophil infiltration is further sustained through positive feedback loops mediated by chemokines such as CXCL8, driving the progression of chronic inflammatory diseases [108]. In particular, dysregulated NET formation plays a central role in chronic inflammation by exposing intracellular components that can act as autoantigens, thereby triggering autoantibody production and perpetuating immune activation [109]. These findings highlight that neutrophils are not merely transient responders but actively participate in sustaining and exacerbating pathological inflammation.

Targeting neutrophils presents unique challenges due to their short lifespan, high motility, and rapid turnover in circulation [110]. Nevertheless, several surface receptors involved in neutrophil recruitment and adhesion have been explored as potential targeting nodes. Chemokine receptors, particularly CXCR2, serve as primary molecular handles for the neutrophil-oriented targeting, as they govern the directed migration of these cells toward inflammatory foci in response to CXCL8. In addition, integrin complexes such as CD11b/CD18 (Mac-1) play critical roles in firm adhesion to the endothelium through interactions with intercellular adhesion molecule-1 (ICAM-1), thereby facilitating neutrophil extravasation into inflamed tissues. Targeting these adhesion molecules enables preferential engagement of activated neutrophils during the transmigration process, particularly within inflamed vascular microenvironments [111]. Beyond chemokine- and integrin-mediated pathways, additional neutrophil-associated surface molecules have also been investigated as potential anchoring sites for targeted delivery. For instance, P-selectin glycoprotein ligand-1 (PSGL-1), a surface glycoprotein involved in selectin-mediated leukocyte rolling, offers robust docking sites for engineered nanocarriers to modulate the initial rolling phase and subsequent vascular adhesion [112]. Collectively, these recruitment- and adhesion-associated receptors expand the targeting repertoire for neutrophil-oriented delivery, although their dynamic expression during inflammation and partial overlap with other leukocyte populations require careful consideration to ensure selective and controlled immune modulation.

In addition to receptors governing neutrophil recruitment and adhesion, receptor systems involved in activation, phagocytosis, and inflammatory signal sensing have also been explored as potential targets for delivery. Fcγ receptors mediate the recognition and internalization of opsonized particles and immune complexes, enabling efficient uptake through receptor-mediated phagocytosis and providing a robust platform for antibody-based targeting strategies [113]. Formyl peptide receptors (FPR1 and FPR2), which belong to the G protein-coupled receptor family, recognize both bacterial-derived N-formyl peptides and endogenous danger-associated molecular patterns, thereby regulating neutrophil chemotaxis and activation [114]. These receptors are particularly attractive for targeting activated neutrophils in inflamed tissues due to their sensitivity to inflammatory gradients.

Compared with macrophage- or T cell-targeting strategies, neutrophil-directed engineered EV systems remain relatively underdeveloped, and most currently available approaches are primarily based on receptor-level targeting concepts rather than experimentally validated EV therapeutics with comprehensive in vitro and in vivo evaluation. This limitation largely reflects the current early stage of neutrophil-specific EV engineering in inflammatory disease models and underscores the need for further experimental validation and therapeutic investigation in this area.

### 3.3. Dendritic Cells

Dendritic cells (DCs) function as professional antigen-presenting cells (APCs) that integrate multifaceted immune cues to orchestrate the transition from innate to adaptive immunity. By continuously surveilling the microenvironment through diverse endocytic and phagocytic pathways, DCs internalize and process exogenous antigens for subsequent presentation to T cells via major histocompatibility complex (MHC) molecules, thereby initiating antigen-specific immune responses. Among specialized subsets, conventional type 1 DCs (cDC1s) are uniquely equipped for cross-presentation, a critical process for initiating cytotoxic T lymphocyte (CTL) responses against intracellular pathogens and aberrant cells [115]. This cross-priming capacity is critically regulated by DC maturation, a process triggered by the recognition of pathogen-associated molecular patterns (PAMPs) or damage-associated molecular patterns (DAMPs). Upon activation, DCs undergo phenotypic maturation characterized by the upregulation of MHC class II, costimulatory molecules such as CD80 and CD86, and the chemokine receptor CCR7, which together facilitate their migration to lymphoid tissues and subsequent T cell activation. This orchestrated expression of homing receptors facilitates DC migration along CCL19/CCL21 gradients to draining lymph nodes, where they prime naïve T cells and orchestrate potent antigen-specific adaptive immune responses.

In addition to their role in immune activation, DCs serve as key regulators of immune homeostasis by maintaining a balance between effector immunity and peripheral tolerance [116]. Under steady-state conditions, immature or semimature DCs continuously sample the microenvironment and migrate to secondary lymphoid organs in a CCR7-dependent manner, where they contribute to the maintenance of tolerance against self-antigens or harmless environmental antigens. These tolerogenic DCs (tolDCs) are characterized by relatively low expression of MHC class II and costimulatory molecules such as CD80, CD86, and CD40, along with the production of anti-inflammatory cytokines, including IL-10 and TGF-β. Functionally, tolDCs suppress excessive immune activation by inducing T cell anergy and promoting the differentiation of naïve T cells into Foxp3^+^ regulatory T cells (Tregs), which actively inhibit inflammatory responses and maintain immune equilibrium. This regulatory capacity is essential for preventing autoimmune pathology and chronic inflammation. However, disruption of these tightly controlled processes, such as impaired DC maturation or migration, can result in either insufficient immune activation, contributing to tumor immune evasion, or loss of tolerance, leading to the development of autoimmune diseases.

Building upon their central immunological role, DCs represent a highly attractive target for selective drug delivery due to their unique ability to dictate the magnitude and direction of antigen-specific immune responses [117,118]. Because DCs serve as the primary gatekeepers of T cell activation and tolerance, the precise delivery of therapeutic agents to these cells enables efficient modulation of downstream adaptive immunity while minimizing off-target effects associated with systemic distribution. In particular, targeted delivery to specific DC subsets allows for controlled induction of either immunostimulatory or tolerogenic responses, depending on the therapeutic context. In this regard, selective targeting of defined DC subsets has emerged as a highly effective strategy to enhance therapeutic precision. In particular, cDC1-specific receptors such as DEC205 and Clec9A have been extensively explored for antigen delivery due to their high expression and efficient internalization capacity [118,119]. Notably, Clec9A is selectively expressed on human CD141^+^ DCs, the functional equivalent of cDC1s, and plays a critical role in regulating CD8^+^ T cell responses [120]. Targeting this receptor enables highly efficient cross-presentation of delivered antigens, resulting in robust activation of antigen-specific CTLs.

In addition to conventional dendritic cell subsets, plasmacytoid dendritic cells (pDCs) have also emerged as important immunomodulatory targets due to their central role in type I interferon production and autoimmune inflammation [121,122]. In particular, aberrant pDC activation has been strongly associated with autoimmune diseases such as systemic lupus erythematosus and rheumatoid arthritis. Although EV-based targeting strategies specifically directed toward pDCs remain relatively limited, modulation of pDC-associated inflammatory signaling represents a promising direction for future engineered EV therapeutics.

Furthermore, beyond protein-based ligands, glycan-dependent interactions represent a critical yet underappreciated mechanism for regulating DC recognition and uptake. C-type lectin receptors, most notably DC-SIGN (dendritic cell-specific intercellular adhesion molecule-3-grabbing non-integrin, CD209), mediate antigen internalization through the specific recognition of carbohydrate structures, particularly mannose- and fucose-containing glycans [67,123]. As a type II transmembrane receptor predominantly expressed on DCs, DC-SIGN functions as a high-capacity endocytic portal that recognizes specific carbohydrate motifs in a calcium-dependent manner. This receptor identifies high-mannose and fucosylated patterns commonly shared by pathogens and apoptotic cells, making it an ideal target for biomimetic nanocarriers decorated with synthetic glycan moieties. Upon ligand engagement, DC-SIGN efficiently shuttles internalized cargo into late endosomal and lysosomal compartments, where antigens are processed for subsequent MHC-mediated presentation. Beyond its role in cargo uptake, DC-SIGN-mediated signaling can actively modulate DC maturation and cytokine profiles, thereby providing a sophisticated mechanism to polarize T cell responses toward either immunity or tolerance depending on the therapeutic objective. However, the presence of lectins on DCs does not necessarily guarantee functional accessibility for glycan-decorated EVs, because endogenous glycans within the glycocalyx can cis-mask lectin-binding sites and limit interactions with external glycan ligands. A previous study showed that the glycan-binding profiles of human blood mononuclear cells, including DCs, do not always correspond directly to the expected repertoire of lectins detected on these cells, particularly in the context of DC-SIGN-associated ligands [124]. Therefore, glycan-based DC targeting should be supported not only by lectin expression analysis but also by functional binding assays that account for lectin accessibility and glycocalyx-dependent masking.

### 3.4. Helper T Cells

CD4^+^ helper T cells are central mediators of the adaptive immune system, responsible for coordinating diverse immune responses through cytokine secretion and direct cell-to-cell interactions. Upon recognizing antigenic peptides presented by MHC class II molecules on professional APCs, naïve CD4^+^ T cells undergo differentiation into distinct effector subsets, including Th1, Th2, Th17, and Tregs, each defined by unique transcriptional programs and cytokine profiles [125]. These subsets exert highly context-dependent roles in immune regulation, collectively maintaining a balance between immune activation and tolerance. In inflammatory diseases, however, dysregulated differentiation skews this balance toward pathogenic responses, leading to persistent immune activation and tissue damage [126]. Among these subsets, Th17 cells play a particularly critical role by promoting neutrophil recruitment and sustained production of pro-inflammatory cytokines such as IL-17, thereby amplifying inflammatory cascades and disrupting tissue homeostasis [127]. Consistent with this, aberrant expansion or functional imbalance of CD4^+^ T cell subsets has been closely associated with the pathogenesis of autoimmune and inflammatory disorders through excessive cytokine secretion and metabolic reprogramming.

Achieving selective delivery to CD4^+^ T cells remains inherently challenging due to their non-phagocytic nature, rapid circulation, and dynamic trafficking behavior across lymphoid and peripheral tissues [128]. To circumvent these physiological barriers, targeting strategies have leveraged lineage-associated surface markers, such as CD4, and the T cell-specific antigen CD7 to facilitate receptor-mediated internalization. As a co-receptor for MHC class II-mediated antigen recognition, CD4 is constitutively expressed across helper T cell subsets, serving as a high-affinity docking site for selective intervention [129,130,131]. Complementarily, CD7 exhibits robust endocytic activity upon ligand engagement, which can be strategically exploited to enhance the cellular uptake of engineered nanocarriers [132]. Functionalizing nanocarriers with high-affinity ligands directed toward these receptors significantly improves targeting precision by prioritizing receptor-mediated endocytosis over non-specific cellular interactions, thereby ensuring the localized delivery of therapeutic payloads within defined T cell populations.

Beyond these primary lineage markers, additional receptor systems associated with T cell activation and differentiation provide important targeting opportunities. The dynamic balance of T cell responses is governed by opposing costimulatory and coinhibitory signals; for instance, CD28 and ICOS are significantly upregulated upon activation to sustain T cell proliferation and survival, thereby enabling the preferential targeting of effector populations [133]. Conversely, the coinhibitory receptor CTLA-4 serves as a pivotal negative regulator by antagonizing CD28-mediated signaling, offering a strategic handle to modulate suppressive immune pathways and restore homeostatic equilibrium [134]. Furthermore, cytokine receptor systems, most notably the IL-2 receptor α-chain (CD25), expand the targeting landscape. As CD25 is preferentially expressed on activated T cells and Tregs, it facilitates the selective engagement of highly proliferative or immunoregulatory subsets, particularly within inflammatory microenvironments where these populations are enriched [135]. Targeting CD25, therefore, enables selective engagement of highly proliferative or immunoregulatory T cell subsets, particularly within inflammatory microenvironments where these populations are enriched.

In addition to CD4^+^ helper T-cell targeting strategies, emerging EV engineering approaches have also explored the modulation of other lymphocyte populations, including CD8^+^ cytotoxic T cells and NK cells, which play critical roles in inflammatory and autoimmune diseases. In particular, engineered EVs targeting CD8^+^ T cells through immune checkpoint-associated receptors such as PD-1 and costimulatory molecules including 4-1BB have demonstrated potential for regulating cytotoxic immune responses and restoring immune homeostasis [95]. Furthermore, EV-based modulation of NK cell activity has attracted increasing interest due to the central role of NK cells in inflammatory tissue injury and immune regulation [96]. Although these approaches remain less extensively investigated compared with macrophage-targeting strategies, they represent promising directions for future engineered EV therapeutics.

**Table 2:** Target immune cells, receptors, ligands, and their biological role.

Immune Cell	Receptor	Biological Role
Macrophage (M1)	CD80/CD86	Co-stimulatory molecules mediating T cell activation and antigen-presenting cell–T cell interaction
Macrophage (M2)	CD206 (MRC1)	Endocytic receptor mediating recognition and clearance of glycosylated pathogens and tissue repair signaling
	CD163	Scavenger receptor involved in hemoglobin–haptoglobin complex clearance and anti-inflammatory macrophage polarization
	MGL (CD301)	C-type lectin receptor recognizing GalNAc-containing glycans and regulating immune tolerance
	FRβ (FOLR2)	High-affinity receptor mediating folate uptake in activated macrophages
	IL-4Rα (CD124)	Cytokine receptor driving M2 macrophage polarization and anti-inflammatory signaling
Dendritic cell	DEC-205 (CD205)	Endocytic receptor mediating antigen uptake and presentation
	Clec9A (CD370)	Receptor recognizing necrotic cell debris and facilitating cross-presentation
	DC-SIGN (CD209)	C-type lectin receptor mediating pathogen recognition and antigen uptake
	LFA-1 (CD11 α/CD18)	Adhesion receptor enabling immune synapse formation between T cells and APCs
Neutrophil	CXCR2 (CD182)	Chemokine receptor controlling neutrophil chemotaxis
	Mac-1 (CD11b/CD18)	Integrin mediating cell adhesion and migration
	PSGL-1 (CD162)	Adhesion receptor enabling leukocyte rolling during inflammation
	FPR	GPCR detecting bacterial peptides and triggering neutrophil activation
CD4+ helper T cell	CD4	Co-receptor that facilitates TCR signaling and antigen recognition via MHC II
	CD7	Surface glycoprotein involved in T-cell activation and intercellular signaling
	CD28	Co-stimulatory receptor essential for full T-cell activation and proliferation
	CTLA-4 (CD152)	Immune checkpoint receptor providing negative regulation of T-cell activation
	ICOS (CD278)	Co-stimulatory receptor promoting T-cell differentiation and cytokine production
	PD-1 (CD279)	Immune checkpoint receptor mediating inhibition of T-cell activation and exhaustion
	CD25 (IL-2Rα)	High-affinity receptor controlling T-cell proliferation and activation
CD8+ cytotoxic T cell	CD8	Co-receptor enhancing recognition of MHC class I–presented antigens
	4-1BB (CD137)	Co-stimulatory receptor promoting T-cell survival, proliferation, and cytotoxic function

## 4. Applications of Engineered Exosomes in Inflammatory Disorders

To validate the functional relevance of engineered EVs, the following disease-specific examples provide compelling experimental evidence linking precise EV–cell engagement to measurable immunomodulatory outcomes (Table 3). These pathological models demonstrate how targeted delivery effectively translates into enhanced uptake by defined cell populations, receptor-dependent signaling modulation, the suppression of pro-inflammatory cytokines, macrophage phenotype reprogramming, and the precise regulation of T cell activation or differentiation, ultimately culminating in the restoration of tissue homeostasis.

### 4.1. Inflammatory Bowel Disease

Inflammatory bowel disease (IBD) is a chronic, relapsing inflammatory disorder of the gastrointestinal tract encompassing Crohn’s disease and ulcerative colitis, and it is characterized by persistent mucosal inflammation, epithelial barrier disruption, and dysregulated immune responses [136,137]. IBD represents a complex and multifactorial disease entity in which genetic susceptibility, environmental triggers, microbial dysbiosis, and aberrant immune activation converge to drive sustained intestinal inflammation. Within the intestinal mucosa, excessive production of reactive oxygen species, disruption of epithelial tight junctions, and imbalances in gut microbiota collectively contribute to the establishment of a pro-inflammatory microenvironment. Despite this multifactorial complexity, these pathological features ultimately converge on the aberrant activation and persistence of immune cell populations within the intestinal lamina propria. Infiltrating macrophages, neutrophils, and effector T cells amplify inflammation through sustained secretion of pro-inflammatory cytokines such as TNF-α, IL-1β, and IL-6, while simultaneously failing to properly resolve inflammation due to impaired regulatory mechanisms. Notably, the dynamic crosstalk between innate and adaptive immune cells further reinforces this pathological loop, driving chronic tissue damage and preventing restoration of intestinal homeostasis.

In this context, targeting key immune cell populations using engineered EVs has emerged as a promising strategy for restoring immune homeostasis. Among these, macrophages, which play central roles in initiating and sustaining intestinal inflammation, have been extensively explored as primary therapeutic targets. Gong et al. developed a Treg-derived EV-based therapeutic platform designed to selectively modulate immune-cell-driven inflammation in IBD [138]. Specifically, the authors engineered Treg-derived exosomes loaded with the antioxidant selenium via sonication and further functionalized their surface with the mitochondria-targeting SS-31 peptide through a matrix metalloproteinase-9 (MMP-9) -cleavable linker (Exo-Se-SS-31). Physicochemically, the engineered Exo-Se-SS-31 exhibited a uniform median diameter of 144.4 nm with a negative zeta potential of −27.17 mV, achieving substantial entrapment efficiencies for both selenium (78.5%) and SS-31 (57.0%). This design enabled inflammation-responsive targeting, as the linker is cleaved within inflamed intestinal tissues where MMP-9 activity is elevated, thereby facilitating site-specific delivery of therapeutic cargo. At the cellular level, Exo-Se-SS-31 exhibited strong affinity toward macrophages, achieving efficient cellular uptake rates of approximately 70% at 24 h and 80% at 48 h, with pronounced colocalization to mitochondria, leading to suppression of mitochondrial reactive oxygen species and restoration of mitochondrial function. Importantly, this system effectively inhibited PANoptosis, a form of inflammatory programmed cell death integrating pyroptosis, apoptosis, and necroptosis, in macrophages stimulated with TNF-α and IFN-γ. In vivo, systemic administration of Exo-Se-SS-31 via tail vein injection in 2.5% dextran sulfate sodium (DSS)-induced colitis models resulted in preferential accumulation within inflamed colon tissues and significant uptake by macrophages in the lamina propria. Therapeutically, treated mice exhibited markedly reduced disease activity index, attenuation of body weight loss, restoration of colon length, and substantial improvement in histological damage compared to control groups. Furthermore, Exo-Se-SS-31 significantly decreased pro-inflammatory cytokine levels, reduced infiltration of CD45^+^ leukocytes and myeloperoxidase-positive neutrophils, and restored epithelial barrier integrity through upregulation of tight junction proteins such as zonula occludens-1 and occludin.

While systemic administration of engineered EVs has proven effective, oral delivery remains the most desirable route for gastrointestinal disorders to ensure high patient compliance; however, the harsh gastric environment presents a major hurdle for protein-based therapeutics. Building upon the concept of macrophage targeting to overcome this challenge, Liu et al. developed an orally deliverable, cytokine-engineered EV platform designed to selectively target inflammatory macrophages in IBD [139]. Specifically, EVs were generated from genetically engineered HEK293T cells to encapsulate IL-10, a potent anti-inflammatory cytokine, followed by surface modification with galactose moieties via DSPE-PEG conjugation to enable macrophage-specific targeting (Gal-IL10-EVs). This functionalization resulted in stable vesicles with a mean diameter of approximately 168.4 nm. To further enable oral delivery, the engineered EVs were encapsulated within a pH-responsive chitosan/alginate hydrogel system formulated at a 3:7 weight ratio (Gal-IL10-EVs (C/A)). This targeting strategy exploits the elevated expression of macrophage galactose-type lectin receptors on activated macrophages within inflamed intestinal tissues, thereby enhancing selective cellular uptake. Mechanistically, Gal-IL10-EVs demonstrated enhanced internalization by macrophages and effectively suppressed intracellular reactive oxygen species levels, while simultaneously inhibiting NF-κB-mediated pro-inflammatory signaling pathways. This resulted in dose-dependent reduction in the secretion of key pro-inflammatory cytokines, including TNF-α, IL-6, IL-12p70, and IL-1β, in LPS-stimulated macrophages, confirming their potent immunomodulatory activity. Importantly, in vivo studies using DSS-induced colitis models demonstrated that orally administered Gal-IL10-EVs (C/A) at a dose of 0.3 mg protein per kg preferentially accumulated in inflamed colon tissues and significantly alleviated disease severity. Treated mice exhibited reduced body weight loss, decreased disease activity index, restoration of colon length, and attenuation of splenomegaly compared to control groups. Histological analysis further revealed substantial improvement in epithelial integrity and reduced inflammatory cell infiltration, while systemic and local cytokine levels were markedly suppressed. Collectively, this study highlights that cytokine-loaded, macrophage-targeted EVs with protective oral delivery systems can effectively modulate immune-cell-driven inflammation and represent a promising therapeutic strategy for IBD.

While modulating the innate immune compartment via macrophage-targeted strategies provides substantial mucosal healing, the chronic and relapsing nature of IBD is fundamentally driven by adaptive immune dysregulation. Therefore, reprogramming pathogenic T-cell responses represents an equally critical and complementary therapeutic frontier. In a recent breakthrough, Oh et al. demonstrated the therapeutic potential of engineered EVs to precisely reprogram these pathogenic T-cell responses [140]. Specifically, bioengineered EVs derived from BMI1/hypoxia-primed Wharton’s jelly mesenchymal stem cells (WJ-MSCs) were designed to simultaneously present PD-L1 on their surface and encapsulate miR-27a-3p (PD-L1/miR-27a-3p EVs). Mechanistically, surface-displayed PD-L1 engages PD-1 on activated T cells, leading to SHP2-mediated dephosphorylation of key T-cell receptor signaling molecules such as ZAP70 and AKT, thereby attenuating TCR-induced calcium influx and suppressing T-cell activation without inducing apoptosis. In parallel, EV-delivered miR-27a-3p directly targets prohibitin 1, a mitochondrial scaffold protein that supports Th17 cell bioenergetics, thereby inhibiting Th17 differentiation while promoting FOXP3^+^ Treg expansion. This coordinated regulation effectively shifts the Th17/Treg balance toward an anti-inflammatory phenotype, which is a central therapeutic objective in IBD. Importantly, these dual-targeting EVs exhibited preferential accumulation in inflamed intestinal tissues, exploited by the high endogenous expression of chemokine receptors. CCR2 and CXCR4, on WJ-MSCs alongside PD-1/PD-L1-dependent interactions. In humanized TNBS-induced colitis models, systemic administration significantly reduced disease severity, as evidenced by decreased body weight loss, improved disease activity index, and preservation of colon structure. Beyond immune modulation, this platform demonstrated excellent safety and tissue compatibility; EV treatment preserved transepithelial electrical resistance (TEER) and tight junction architecture in both Caco-2 monolayers and IBD patient-derived 3D colonoid models without inducing cytotoxicity. These findings highlight the sophisticated advantage of EV-based therapeutics in achieving precise, localized adaptive immune reprogramming while maintaining intestinal epithelial homeostasis.

Collectively, these studies illustrate that next-generation engineered EVs can achieve highly precise immune modulation in IBD by integrating cell-specific targeting, surface engineering, and intracellular reprogramming strategies. This multi-layered approach represents a significant advancement beyond conventional cytokine-neutralizing therapies, offering a scalable, cell-free platform capable of restoring immune balance and intestinal homeostasis while minimizing systemic immunosuppression.

Overall, these studies highlight that engineered EVs can achieve precise immune modulation in IBD through the integration of macrophage targeting, adaptive immune re-programming, and localized therapeutic delivery. In particular, macrophage-targeting strategies demonstrated the most consistently validated in vivo therapeutic efficacy, whereas approaches targeting the Th17/Treg axis provided promising potential for long-term immune homeostasis. Nevertheless, variability in targeting ligands, cargo composition, and delivery routes continues to complicate direct comparison between studies.

### 4.2. Rheumatoid Arthritis

Rheumatoid arthritis (RA) is a chronic, systemic autoimmune inflammatory disease primarily affecting synovial joints, characterized by persistent synovial inflammation, pannus formation, and progressive destruction of cartilage and bone [141]. Within the inflamed synovium, macrophages, DCs, and fibroblast-like synoviocytes collectively contribute to the establishment of a highly inflammatory microenvironment. In particular, pro-inflammatory macrophages play a central role in initiating and amplifying disease progression through the secretion of TNF-α, IL-1β, and IL-6, while also promoting T-cell activation via costimulatory signaling [142]. Concurrently, pathogenic Th1 and Th17 responses are expanded, whereas regulatory T-cell function remains insufficient, resulting in failure to restore immune tolerance. This imbalance between pro-inflammatory and regulatory immune pathways ultimately drives chronic joint destruction, positioning RA as a prototypical immune-cell-driven inflammatory disease.

Macrophage-targeted EV engineering strategies have demonstrated strong therapeutic potential in RA by directly modulating the dominant inflammatory cell population. In one representative study, You et al. developed a metabolically engineered EV platform designed to selectively target and reprogram pro-inflammatory macrophages within inflamed synovial tissues [143]. In this approach, adipose-derived stem cells were first metabolically glycoengineered using Ac4ManNAz to introduce azide functional groups onto the cell surface via the sialic acid biosynthetic pathway. Subsequently, dibenzocyclooctyne-conjugated dextran sulfate (DBCO-DS) was covalently attached through copper-free bioorthogonal click chemistry, enabling the presentation of dextran sulfate as a targeting ligand on the exosomal membrane (DS-EXOs). Dextran sulfate was specifically selected due to its high binding affinity toward scavenger receptor class A (SR-A), which is abundantly overexpressed on activated M1 macrophages within inflamed synovial tissues. This surface engineering yielded highly stable vesicles (136.3 ± 9.0 nm) that present approximately 20,811 ± 4261 DS molecules per EXO without compromising their intrinsic structural integrity or therapeutic cargo. Functionally, DS-EXOs retain the endogenous therapeutic cargo of mesenchymal stem cell–derived exosomes, particularly microRNAs such as let-7b-5p and miR-24-3p, which are key regulators of macrophage polarization pathways. These microRNAs were shown to modulate multiple signaling cascades, including MAPK, PI3K–Akt, Wnt, and JAK–STAT pathways, ultimately promoting the phenotypic transition from pro-inflammatory M1 macrophages to anti-inflammatory M2 macrophages. Importantly, the engineered EVs not only enhanced M1-to-M2 polarization but also inhibited reverse polarization under inflammatory conditions, thereby stabilizing the anti-inflammatory macrophage phenotype. Mechanistic inhibition studies using microRNA antagonists further confirmed that the therapeutic efficacy of these EVs is critically dependent on these exosomal microRNAs, underscoring the importance of preserving intrinsic cargo functionality during surface engineering. At the therapeutic level, systemic administration in collagen-induced arthritis models resulted in significant attenuation of disease progression, including reductions in clinical arthritis scores, paw swelling, inflammatory cytokine levels such as TNF-α, IL-1β, and IL-6, and histological markers of synovial inflammation, cartilage degradation, and bone erosion. Notably, comparable therapeutic efficacy was achieved at approximately 10-fold lower doses relative to unmodified exosomes, highlighting the efficiency gains conferred by targeted delivery. Additionally, treatment increased levels of anti-inflammatory cytokines such as IL-4 and IL-10 while reducing pro-inflammatory mediators and Th17-associated responses, indicating a broader immunomodulatory effect on the synovial microenvironment. Collectively, this metabolically engineered EV system, integrating Ac4ManNAz-mediated glycoengineering and DBCO-DS–based targeting, provides a highly effective platform for precise macrophage targeting, immune reprogramming, and restoration of inflammatory homeostasis in RA.

While mesenchymal-stem-cell-derived exosomes offer intrinsic therapeutic cargo, harnessing exosomes derived directly from immune cells presents a highly biomimetic alternative that inherently homes to inflammatory lesions. Building upon macrophage-targeted EV engineering strategies, Yan et al. developed a folic acid-functionalized, macrophage-derived exosome platform encapsulating dexamethasone sodium phosphate (FPC-Exo/Dex) to actively target inflamed joints enriched with folate receptor β-overexpressing activated macrophages [144]. In this system, exosomes secreted by RAW 264.7 macrophages were first loaded with dexamethasone via electroporation, achieving a notable drug loading efficiency of 18.81%, followed by the post-insertion of a folic acid–PEG–cholesterol conjugate into the exosomal membrane. The incorporation of PEG contributed to enhanced colloidal stability and prolonged systemic circulation, while folic acid enabled receptor-mediated targeting toward inflammatory macrophages. Physicochemical characterization revealed a uniform nanoscale size distribution of approximately 128.4 nm with a negative surface charge, −22.73 mV. Furthermore, the platform exhibited pH-responsive sustained drug release behavior, releasing nearly 80% of its payload within 16 h under mildly acidic conditions (pH 6.0) mimicking inflamed tissues, compared to less than 60% at physiological pH. Functionally, this engineered platform demonstrated significantly enhanced cellular uptake in LPS-activated macrophages compared to non-modified exosomes and liposomal controls, confirming effective targeting specificity. This translated into superior anti-inflammatory activity through marked suppression of pro-inflammatory cytokines such as TNF-α and IL-1β, alongside increased production of the anti-inflammatory cytokine IL-10. Importantly, in vivo biodistribution studies in collagen-induced arthritis models showed that FPC-Exo/Dex exhibited preferential accumulation and prolonged retention within inflamed joints, consistent with both enhanced permeability-driven passive targeting and folate receptor-mediated active targeting mechanisms. Therapeutically, systemic administration resulted in substantial attenuation of disease progression, as evidenced by reduced arthritis scores, decreased paw swelling, and restoration of body weight, accompanied by significant improvements in histopathological outcomes, including reduced synovial hyperplasia, inflammatory cell infiltration, cartilage degradation, and bone erosion.

In this context, advanced combinatorial engineering approaches have been developed to enable coordinated modulation of both macrophage function and downstream adaptive immune responses. Yang et al. developed CD80 antibody and methotrexate co-engineered extracellular vesicles (anti-CD80-MTX-EVs) to selectively target pro-inflammatory CD80^+^ macrophages and simultaneously modulate macrophage–T cell interactions [91]. In this system, DSPE-PEG–conjugated anti-CD80 antibodies were incorporated into the plasma membrane of human umbilical cord mesenchymal stem cells, enabling the generation of surface-functionalized EVs during vesicle biogenesis. Concurrently, methotrexate was introduced into the parental cells, resulting in efficient encapsulation within EVs and formation of dual-functional anti-CD80-MTX-EVs during natural vesicle biogenesis. This cellular engineering process yielded functionalized EVs with an average diameter of approximately 450 nm and an MTX encapsulation concentration of approximately 0.4 µg/mL. Functionally, these engineered EVs exhibited significantly enhanced uptake by CD80^+^ bone marrow-derived macrophages compared to non-modified EVs, leading to reduced CD80 expression and a shift toward an anti-inflammatory phenotype. Mechanistically, RNA-sequencing revealed that treatment with anti-CD80-MTX-EVs promoted upregulation of TGF-β signaling pathways in macrophages, as evidenced by increased expression of Tgfb2, Tgfb3, Bmbpr2, and Smad5, which are critical mediators of immune tolerance. This macrophage reprogramming subsequently induced robust expansion of Tregs while suppressing Th1 cell differentiation, highlighting coordinated regulation of both innate and adaptive immune responses. At the therapeutic level, systemic administration in collagen-induced arthritis models resulted in significant attenuation of disease progression, including reductions in arthritis scores, paw swelling, and pro-inflammatory cytokines such as TNF-α and IFN-γ, alongside increased levels of anti-inflammatory mediators, including IL-10 and TGF-β. Histological analysis further confirmed reduced inflammatory cell infiltration and preservation of joint architecture. Notably, biodistribution studies demonstrated enhanced accumulation of anti-CD80-MTX-EVs in inflamed joints compared to non-targeted EVs, indicating effective in vivo targeting of pathogenic macrophage populations. Beyond RA, similar therapeutic efficacy was observed in periodontitis models, where treatment reduced bone loss and inflammatory cytokine expression while promoting immune tolerance.

Complementing these antibody-directed and combinatorial targeting strategies, surface-modified EV platforms leveraging extracellular matrix–mimetic ligands have also been explored to enhance inflammatory site accumulation and macrophage-specific uptake in a more biologically integrative manner. In a recent study, Zhang et al. developed hyaluronic acid and PEG-modified BMSC-derived EVs (Cur@EXs-PH) to selectively target CD44-overexpressing pro-inflammatory macrophages while improving systemic stability [145]. In this system, hyaluronic acid served as a targeting ligand for CD44, which is highly upregulated on activated M1 macrophages, while PEG modification reduced opsonization and prolonged circulation time. Curcumin was subsequently encapsulated via sonication, achieving a notable encapsulation efficiency of 29.78%, to provide anti-inflammatory and tissue-reparative effects. Physicochemical characterization confirmed that PH modification preserved the EV structure, resulting in stable nanoparticles of approximately 131.58 nm with a shifted zeta potential, from −28.36 mV to −14.68 mV, that maintained stability for over 7 days. Functionally, these EVs exhibited significantly enhanced uptake in M1 macrophages and preferential accumulation at inflammatory sites compared to non-modified EVs, confirming effective targeting specificity. Mechanistically, Cur@EXs-PH suppressed NLRP3 inflammasome activation and reduced pro-inflammatory cytokine production, while simultaneously promoting macrophage repolarization toward an anti-inflammatory M2 phenotype, achieving an M2 proportion of nearly 43.87% in vitro. At the therapeutic level, treatment in both acute and chronic inflammation models, including spinal cord injury and collagen-induced arthritis, resulted in marked attenuation of inflammation, reduced tissue damage, and improved functional recovery, accompanied by decreased levels of TNF-α, IL-1β, and IL-6 and increased anti-inflammatory mediators such as TGF-β. Collectively, this study highlights that extracellular matrix–inspired ligand functionalization combined with pharmacological cargo loading provides an effective strategy for enhancing macrophage targeting, improving in vivo stability, and achieving robust immunomodulatory effects in inflammatory diseases.

While previous strategies primarily focused on macrophage reprogramming and cytokine modulation, recent advances have further extended EV-based platforms toward the induction of antigen-specific immune tolerance by directly targeting and selectively eliminating pathogenic T cell populations. Wu et al. developed an M2 macrophage-derived exosome platform (M2CPR) incorporating copper sulfide nanoparticles, citrullinated peptide antigens, and rapamycin, designed to selectively eliminate pathogenic T cells while simultaneously promoting immune tolerance [146]. Leveraging the inherent inflammatory homing capacity of M2-derived EVs, this system preferentially accumulated within inflamed synovial tissues and ectopic lymphoid structures, where activated T cells are highly enriched. Mechanistically, the 5 nm copper sulfide nanoparticles released from M2CPR induced cuproptosis in activated T cells, a mitochondria-dependent cell death pathway driven by copper accumulation and enhanced oxidative phosphorylation activity. This metabolic vulnerability enabled preferential elimination of pathogenic effector T cells by promoting the oligomerization and degradation of lipoylated TCA cycle enzymes, while largely sparing non-activated immune populations. Importantly, clearance of apoptotic T cell fragments by macrophages triggered robust secretion of TGF-β, which promoted differentiation of tolDCs, together with the mTOR inhibitor, rapamycin. These cells subsequently presented citrullinated peptide antigens under low co-stimulatory conditions, driving naïve T cell differentiation into Tregs and establishing a self-amplifying immune tolerance loop. At the therapeutic level, systemic administration of M2CPR in collagen-induced arthritis models resulted in significant suppression of disease progression, characterized by reduced pro-inflammatory cytokines such as TNF-α, IL-1β, and IL-17, alongside increased levels of anti-inflammatory mediators including IL-10 and TGF-β. Notably, this platform achieved sustained immune tolerance even after an additional two-month observation phase following 52 days of treatment, as evidenced by persistent Treg populations and reduced disease relapse, highlighting its strong potential for durable and long-term disease control.

These findings suggest that EV-based therapeutics in RA are evolving from simple anti-inflammatory delivery systems toward increasingly sophisticated platforms capable of coordinated innate and adaptive immune regulation. In particular, macrophage-targeting approaches demonstrated highly reproducible in vivo selectivity and inflammatory suppression, whereas antigen-specific tolerance strategies showed greater potential for durable therapeutic effects and long-term disease control.

### 4.3. Other Inflammatory Disorders

Beyond gastrointestinal and joint-specific inflammatory diseases, engineered EV-based therapeutic strategies have also demonstrated significant potential in a broader spectrum of immune-mediated inflammatory disorders, including those affecting the central nervous system. Multiple sclerosis (MS), a prototypical autoimmune demyelinating disease of the CNS, is characterized by immune-mediated destruction of myelin sheaths, leading to progressive neurodegeneration and neurological dysfunction. Although current therapeutic approaches primarily focus on suppressing peripheral immune responses, they remain insufficient to promote effective remyelination and functional recovery, highlighting the need for advanced delivery systems capable of targeting the CNS microenvironment [147]. In this context, Zhai et al. developed a brain-targeted engineered EV platform for the efficient delivery of brain-derived neurotrophic factor (RVG/BDNF-Exo), aimed at promoting remyelination in MS [148]. Specifically, EVs were generated from genetically engineered HEK293T cells overexpressing an RVG-Lamp2b fusion protein to confer neuron-targeting capability, while simultaneously loading BDNF at both the mRNA and protein levels through intracellular engineering. Physicochemically, these engineered vesicles maintained a uniform size of approximately 100 nm and achieved a remarkably high BDNF encapsulation concentration of 65.86 pg/mg of exosomal protein. This dual-engineered system enabled enhanced delivery specificity to the CNS rather than direct targeting of infiltrating immune cells. Notably, the use of intranasal administration allowed the engineered EVs to bypass the blood–brain barrier and achieve significantly higher accumulation in brain tissues compared to intravenous delivery, which is typically limited by hepatic and splenic clearance. RVG-mediated targeting facilitated efficient EV distribution across key brain regions, including the olfactory bulb, cortex, and thalamus, while EV-delivered BDNF activated the TrkB–MAPK/Erk signaling pathway, thereby promoting differentiation of oligodendrocyte precursor cells into mature oligodendrocytes and facilitating myelin regeneration. At the therapeutic level, a two-week intranasal administration of RVG/BDNF-Exo in cuprizone-induced demyelination models resulted in significant improvement in motor coordination, increased numbers of myelinated axons, reduced g-ratio values, and enhanced expression of myelin-associated proteins such as myelin basic protein and myelin oligodendrocyte glycoprotein.

Extending these EV-based therapeutic strategies to pulmonary inflammatory diseases, approaches that combine tissue-specific targeting with anti-inflammatory cargo delivery have also demonstrated significant therapeutic potential, even in the absence of direct immune cell targeting. In a representative study, Kim et al. developed engineered exosomes for inhalation-based pulmonary delivery of a RAGE-binding peptide and curcumin (RBP-exo/Cur), designed to modulate inflammation within lung tissues affected by acute lung injury [149]. In this system, the RAGE-binding peptide was genetically fused to the exosomal membrane protein Lamp2b, enabling its display on the EV surface, while the hydrophobic anti-inflammatory agent curcumin was subsequently incorporated into the exosomal lipid bilayer via passive loading. This engineering process yielded uniform vesicles approximately 30–35 nm in size, and 36.4 ± 4.2% of the initially added curcumin was successfully incorporated into RBP-exo. This design allowed simultaneous targeting of RAGE-overexpressing inflammatory lung cells and efficient intracellular delivery of curcumin, with over 50% of the curcumin released within 2 h under physiological conditions. These engineered EVs exhibited enhanced cellular uptake and significantly improved anti-inflammatory efficacy in LPS-activated macrophages compared to free curcumin or non-modified exosomes, as evidenced by marked reductions in pro-inflammatory cytokines including TNF-α, IL-6, and IL-1β, along with effective suppression of intracellular reactive oxygen species. Notably, in vivo administration via intratracheal instillation in acute lung injury models resulted in preferential accumulation of EVs within lung tissues, particularly in type I alveolar epithelial cells, where RAGE expression is highly elevated. Therapeutically, treatment with RBP-exo/Cur significantly reduced inflammatory cytokine levels in both lung tissue and bronchoalveolar lavage fluid, attenuated immune cell infiltration, and improved histopathological outcomes, including reduced tissue damage and edema. These findings suggest that EV-based pulmonary delivery systems can effectively target inflamed tissue microenvironments and achieve robust anti-inflammatory effects through combined ligand-mediated targeting and drug delivery, even when direct immune cell targeting is not the primary mechanism. Building upon these tissue-targeting strategies, liver-specific delivery of engineered EVs has also been achieved through biomaterial-based surface modification approaches. In a representative study, Tamura et al. demonstrated that exosomes can be effectively redirected to injured liver tissue via simple surface modification with cationized pullulan, a polysaccharide known to interact with the asialoglycoprotein receptor expressed on hepatocytes. Unlike genetic engineering strategies, this approach relies on electrostatic interactions between the positively charged pullulan derivative and the negatively charged exosomal membrane, enabling rapid and scalable surface functionalization without altering the intrinsic biological properties of EVs. As a result, modified EVs exhibited significantly enhanced cellular uptake in HepG2 cells through receptor-mediated endocytosis, while inhibition of this receptor markedly reduced internalization, confirming the specificity of this targeting mechanism. Importantly, in vivo studies using a concanavalin A-induced liver injury model revealed that pullulan-modified EVs preferentially accumulated in liver tissue, with a substantial increase in distribution beyond Kupffer cells to include hepatocytes, indicating a shift from passive macrophage uptake toward active receptor-mediated targeting. This enhanced accumulation translated into significantly improved therapeutic outcomes, including reduced necrotic areas, decreased plasma alanine aminotransferase levels, and suppression of pro-inflammatory cytokines such as IL-1β and TNF-α, alongside upregulation of anti-inflammatory mediators, including IL-10 and hepatocyte growth factor. Furthermore, the treatment promoted Treg expansion, suggesting a broader immunomodulatory effect beyond localized tissue repair.

Extending EV-based targeting strategies to joint-specific inflammatory diseases, highly specialized cell-targeting approaches have been developed to overcome the unique structural and physiological barriers of cartilage tissue. Osteoarthritis (OA), a chronic degenerative joint disease characterized by progressive cartilage breakdown and extracellular matrix degradation, presents a particularly challenging microenvironment due to its dense, avascular structure that severely limits drug penetration and retention. In this context, Liang et al. engineered chondrocyte-targeting exosomes for the efficient delivery of microRNA-140 (CAP-exo/miR-140), a key regulator of cartilage homeostasis [150]. Specifically, a chondrocyte-affinity peptide was genetically fused to Lamp2b, enabling selective surface display on dendritic cell-derived EV membranes and facilitating targeted interaction with chondrocytes. Through electroporation, miR-140 was encapsulated with an approximate 60% loading efficiency, resulting in stable vesicles ranging from 40 to 200 nm in diameter. Equipped with this targeting moiety, the engineered EVs were capable of penetrating the dense cartilage extracellular matrix and preferentially accumulating within chondrocytes, overcoming one of the major barriers in OA drug delivery. Functionally, CAP-modified EVs exhibited significantly enhanced cellular uptake and selective delivery of miR-140 to chondrocytes compared to non-targeted EVs, leading to substantial upregulation of intracellular miR-140 levels and subsequent downregulation of matrix-degrading enzymes such as MMP-13 and ADAMTS-5, which are central mediators of cartilage degradation. Importantly, in vivo intra-articular administration demonstrated that CAP-engineered EVs were effectively retained within the joint cavity with minimal off-target distribution, while enabling deep penetration into the middle zones of cartilage tissue, a region typically inaccessible to conventional therapeutics. This enhanced localization translated into pronounced therapeutic benefits, including preservation of cartilage structure, restoration of proteoglycan content, and significant reduction in histological OA severity scores.

Expanding the horizon of EV-based therapies from cartilage repair to broader skeletal inflammatory conditions, innovative biomimetic approaches have emerged to tackle the complex multicellular environments driving bone resorption. In this context, Cai et al. developed a bacteria-derived EV-mimicking “trojan horse” nanoparticle system for selective targeting of pro-inflammatory M1 macrophages [151]. Specifically, outer membrane vesicles derived from *Escherichia coli* were used to coat gold nanocages (AuNC-OM), enabling inheritance of PAMPs from bacterial membranes. This design enables multivalent interactions with phagocytosis-related receptors, including CD64 and CD14, which were expressed at approximately 4-fold and 3.7-fold higher levels on M1 macrophages compared to their M2 counterparts. Mechanistically, the outer membrane vesicle-coated nanoparticles exploit innate immune recognition pathways, where bacterial membrane components such as LPS and outer membrane protein A facilitate receptor-mediated uptake. Importantly, in a physiologically relevant three-dimensional dynamic co-culture system that mimics macrophage heterogeneity, AuNC-OM demonstrated preferential uptake by M1 macrophages over M2 macrophages, highlighting the importance of competitive cellular interactions in evaluating targeting specificity. For therapeutic application, the nano-particles were loaded with dexmedetomidine (AuNC-OM-dex, 10~15% drug loading capacity), enabling controlled drug release upon near-infrared irradiation. This system effectively suppressed NF-κB signaling by reducing the p-p65/p65 ratio by nearly 50% and decreasing nuclear translocation, which subsequently reduced the production of IL-1β and TNF-α and attenuated M1 macrophage polarization markers. Importantly, in vivo evaluation using an inflammation-induced bone resorption model demonstrated that AuNC-OM-dex preferentially accumulated in macrophages and significantly alleviated inflammation-driven tissue damage. Treated mice exhibited reduced osteoclast formation, a ~15% increase in the bone volume/tissue volume ratio, indicating preservation of bone structure, and substantially decreased expression of inducible nitric oxide synthase in the defect area.

Together, these examples demonstrate the remarkable versatility of engineered EV platforms across diverse inflammatory microenvironments and tissue-specific pathological settings. In particular, localized delivery strategies such as intranasal, inhalation-based, and intra-articular administration improved tissue selectivity and therapeutic efficacy while minimizing systemic off-target distribution. Nevertheless, differences in engineering strategies and efficacy evaluation methods continue to limit direct comparison across studies.

**Table 3:** Summary of disease-specific targeting strategies for engineered EVs.

Inflammatory Disease	EV Origin	Target Cell	Ligand	Engineering Strategy	Key Targeting/ Efficacy Outcome	Ref.
Inflammatory Bowel Disease	Treg	Macrophage	SS-31 (mitochondria-targeting tetrapeptide)	Chemical surface modification (thiol–maleimide conjugation)	Enhanced accumulation in the inflamed colon and macrophage uptake; mitochondria-targeted delivery	[138]
	HEK293T cell	Macrophage	Galactose	Physical membrane modification (DSPE lipid—EV membrane hydrophobic insertion of galactose)	Galactose-mediated macrophage uptake increased compared with naïve EVs; C/A hydrogel protected EVs in SGF and enabled colon-responsive release; the strongest colon accumulation at 24 h after oral administration	[139]
	Wharton’s jelly mesenchymal stem cell	Activated T cell	PD-L1	Genetic engineering (Lentiviral transduction for PD-L1 surface display)	Preferential accumulation in inflamed intestine via CCR2/CXCR4-associated homing; enhanced uptake by colonic CD4^+^ T cells; suppression of TCR signaling through SHP2 activation and reduced p-ZAP70/p-AKT; reduced Th1/Th17 polarization and increased FOXP3^+^ Treg induction	[140]
Rheumatoid Arthritis	Adipose-derived stem cell (ADSC)	Macrophage	Dextran sulfate (DS)	Metabolic glycoengineering and copper-free click chemistry (Ac4ManNAz and DBCO-DS)	Enhanced uptake into activated RAW264.7 macrophages and BMDMs via SR-A-mediated endocytosis; 23.9-fold higher uptake than bare EXOs in activated RAW264.7 cells at 3 h; preferential accumulation in inflamed joints with 1.52-fold higher fluorescence intensity at 1 h post-injection; colocalization with SR-A^+^ synovial macrophages in vivo	[143]
	RAW 264.7 Macrophage	Macrophage	Folic acid (FA)	Physical membrane modification (FA–PEG–Chol lipid post-insertion via hydrophobic interaction)	Enhanced uptake into LPS-activated RAW264.7 macrophages; stronger accumulation and prolonged retention in inflamed joints compared with Lip/Dex and Exo/Dex	[144]
	Human umbilical cord MSC (hUCMSC)	Pro-inflammatory CD80^+^ macrophages	anti-CD80 antibody	Pre-secretion modification (Parental cell membrane functionalization with DSPE-PEG-anti-CD80)	Enhanced macrophage uptake and inflamed tissue accumulation; reduced CD80^+^ macrophages; increased Treg induction and IL-10 secretion	[91]
	Bone marrow MSC (BMSC)	Pro-inflammatory M1 macrophages (CD44^+^)	Hyaluronic acid (HA)	Chemical surface modification (Thiol-based conjugation of HA-PEG-SH)	Enhanced uptake by M1 macrophages and increased accumulation at inflammatory sites; prolonged circulation half-life (~8.5h); Cur@EXs-PH showed > 2-fold higher inflammatory-site accumulation than Cur@EXs in CIA mice	[145]
	M2 Macrophage	Activated T cells (in Ectopic Lymphoid Structures)	Inherent M2 surface receptors	Cargo engineering (Sonication-mediated loading of CuS NPs and Rapamycin, co-incubation with CitP)	Hind paw accumulation by IVIS imaging; liver secondary accumulation	[146]
Multiple Sclerosis (MS)	Panax ginseng root	Oligodendrocyte/Neurons in CNS	RVG (Rabies virus glycoprotein) peptide	Genetic engineering (RVG-Lamp2b fusion protein display on exosome membrane)	RVG-modified exosomes showed enhanced brain accumulation after intranasal delivery, with preferential distribution in the olfactory bulb, thalamus, and cortex; IV delivery mainly accumulated in the liver/spleen	[148]
Acute Lung Injury	HEK293 cell	RAGE^+^ alveolar epithelial cells	RBP (RAGE-binding peptide)	Genetic engineering (RBP-Lamp2b fusion protein display on exosome membrane)	RBP-exo/Cur showed enhanced intracellular curcumin uptake in LPS-activated cells via RAGE interaction and preferential co-localization with type I alveolar epithelial cells in vivo	[149]
Osteoarthritis	Dendritic cell	Chondrocytes	CAP (chondrocyte-affinity peptide)	Genetic engineering (CAP peptide–Lamp2b fusion protein display on exosome membrane)	CAP-exosomes selectively entered chondrocytes, penetrated deep cartilage regions, and remained confined within joints with minimal systemic diffusion	[150]
Inflammatory Bone Resorption	*Escherichia coli* (Outer membrane vesicles)	Pro-inflammatory M1 macrophages	PAMPs (e.g., LPS, ompA) from the bacterial membrane	Membrane coating (OMV coating onto gold nanocages)	AuNC-OM selectively accumulated in M1 macrophages through CD64/CD14-mediated uptake and showed ~3-fold higher uptake in M1 macrophages within the 3D multicellular co-culture system	[151]

## 5. Discussion and Future Perspectives

Despite substantial progress in EV-based targeting strategies, several critical limitations continue to hinder their clinical translation [152]. A primary challenge lies in the lack of precise and reproducible control over ligand presentation on the EV surface [34]. Importantly, the number and distribution of functional moieties on EV membranes are inherently heterogeneous, including variations in reactive groups such as amines, thiols, and carboxyl groups, depending on the parental cell type, cellular state, and even between production batches derived from the same source. Moreover, this variability is not only observed across different batches but can also exist within a single batch, making it inherently difficult to achieve uniform surface functionalization. As a result, chemical conjugation approaches that rely on these reactive groups face fundamental limitations in enabling precise and quantitative control over ligand density and orientation, often leading to heterogeneous surface coverage and unpredictable targeting performance. While genetic engineering enables endogenous expression of targeting moieties, it is also constrained by variable incorporation efficiency into EV membranes, resulting in inconsistent ligand display and limited control over functional accessibility. Collectively, these limitations highlight the need for next-generation engineering strategies that enable precise, site-specific, and quantitatively controlled ligand incorporation to ensure uniformity and scalability in EV design.

A second major barrier arises from the intrinsic complexity and dynamic nature of the immune microenvironment. Although receptor–ligand-based targeting has demonstrated promising specificity in vitro and in simplified in vivo models, many commonly targeted markers, including CD80, CD86, CXCR2, and other inflammation-associated chemokine receptors, are not uniquely expressed by a single immune cell subset. Instead, their expression is highly context-dependent and dynamically regulated in response to inflammatory cues, leading to significant overlap across immune populations. This biological redundancy can result in off-target uptake and diminished targeting precision. Moreover, spatiotemporal heterogeneity within diseased tissues further complicates targeting outcomes, as shifts in cytokine gradients, immune cell composition, and metabolic states can alter receptor availability over time [153]. These limitations highlight the need for the continued identification and validation of more selective and stable surface markers that enable precise targeting of specific immune cell subsets and lineages, along with a more refined understanding of immune cell heterogeneity under pathological conditions. In this context, tissue-dependent factors such as vascular architecture, local immune composition, and stromal barriers are also likely to influence EV biodistribution and cellular uptake, suggesting that targeting strategies may benefit from being tailored not only to specific cell types but also to the tissue environments in which they reside. To address these limitations, future EV platforms may benefit from multiparametric targeting strategies that combine multi-receptor recognition with microenvironment-responsive activation. For example, dual-receptor recognition systems could improve specificity by requiring simultaneous or sequential engagement of two disease-associated markers, while pH-, protease-, redox-, or enzyme-responsive designs could keep EVs relatively inactive in circulation and activate ligand exposure or cargo release selectively within inflamed tissues. These sequential and context-dependent targeting principles may reduce off-target uptake caused by overlapping marker expression and provide a more precise strategy for navigating the spatiotemporal heterogeneity of inflammatory microenvironments.

Several EV-based therapeutics have entered early-phase clinical evaluation, highlighting the growing translational interest in EV platforms. However, most engineered EV-targeting strategies discussed in this review remain at the preclinical stage. In addition to biological and targeting-related challenges, large-scale production of engineered EVs remains a significant bottleneck for clinical translation. Notably, EV-based therapeutic platforms are increasingly expanding beyond conventional mammalian cell culture-derived sources to include alternative sources such as milk-derived EVs and plant-derived EV-like nanovesicles, which may offer advantages in scalability and source availability [154,155]. In parallel, advances in isolation and purification techniques have improved yield, reproducibility, and processing throughput, providing a foundation for more feasible large-scale production. However, introducing targeting functionalities at scale remains particularly challenging. Surface engineering approaches, including genetic modification and chemical conjugation, are often difficult to integrate into standardized, high-throughput manufacturing processes due to variability in modification efficiency, instability of functionalized components, and challenges in maintaining batch consistency. These limitations are further compounded by the intrinsic heterogeneity of EV populations, which becomes increasingly difficult to control during scale-up. As a result, while bulk EV production is becoming more accessible, the scalable generation of uniformly engineered, target-specific EVs remains limited. In addition to manufacturing scalability, substantial heterogeneity in EV composition, isolation, and characterization remains a major challenge for clinical translation. EV properties and therapeutic efficacy may vary depending on the parental cell source, purification methods, and storage conditions, thereby affecting EV purity, biological activity, and batch-to-batch reproducibility. Addressing these challenges will require the development of robust and standardized manufacturing platforms that enable precise surface functionalization, quality control, and reproducibility at scale, highlighting the urgent need for innovation in EV production and engineering processes. Several EV-based therapeutics have entered early-phase clinical evaluation, highlighting the growing translational interest in EV platforms. However, most engineered EV-targeting strategies discussed in this review remain at the preclinical stage.

Collectively, these advances underscore the growing therapeutic and translational potential of EVs in inflammatory diseases, largely driven by their innate ability to modulate immune functions and reshape pathological microenvironments. Translating this potential into clinical-grade efficacy requires not only efficient accumulation at inflamed sites but also precise engagement with specific immune cell subsets. Current limitations in targeting precision often lead to non-selective distribution, which dilutes therapeutic potency and increases the risk of off-target effects. In this context, surface engineering strategies can substantially influence the pharmacokinetic behavior and biodistribution of EVs in vivo. Modifications such as PEGylation or lipid insertion may prolong systemic circulation by reducing opsonization and mononuclear phagocyte system-mediated clearance, whereas receptor-targeting ligands can enhance selective accumulation in specific tissues or immune cell populations. In addition, alterations in membrane composition and surface charge may affect tissue penetration, cellular uptake, and intracellular trafficking. However, excessive surface functionalization may also alter EV tropism or increase hepatic and splenic sequestration, highlighting the need to carefully balance targeting specificity with systemic biodistribution. Therefore, understanding how individual engineering strategies influence EV fate in vivo will be essential for the rational design and clinical translation of engineered EV therapeutics.

To overcome these barriers, next-generation EV platforms must move beyond static ligand–receptor interactions toward sophisticated, programmable, and scalable engineering strategies. Integrating dynamic modalities, including hierarchical recognition and microenvironment-adaptive responses, will be essential to navigate the spatiotemporal complexity of immune environments. Collectively, such advances, supported by rigorous mechanistic and translational investigations, will be critical for enabling precise immune modulation and unlocking the full therapeutic potential of engineered EVs in inflammatory diseases.

## Figures and Tables

**Table 1:** Comparative summary of EV engineering strategies for cell-specific delivery.

Engineering Method	Main Principle	Key Advantages	Translational Considerations
Genetic engineering	Donor cells are engineered to express ligand-fusion proteins that are incorporated into EV membranes during biogenesis	Stable ligand display, controlled orientation, biologically integrated surface presentation	Requires stable producer cell lines, genetic construct validation, and careful quality control
Chemical conjugation	Targeting ligands are covalently attached to reactive groups on isolated EV surfaces	Broad ligand compatibility, strong covalent linkage, post-isolation modularity	Requires optimization of reaction conditions, ligand density, residual reagent removal, and batch reproducibility
Lipid post-insertion	Ligand-conjugated lipids are inserted into the EV membrane through hydrophobic interactions	Simple process, relatively mild conditions, favorable preservation of native EV membrane proteins	Suitable for scalable formulation but requires stability testing and characterization of ligand retention
Membrane fusion	EVs are fused with synthetic liposomes or lipid vesicles to generate hybrid vesicles	Allows tuning of membrane charge, fluidity, stability, and cargo-loading capacity	Requires robust process control, hybrid vesicle characterization, and safety evaluation of added lipid components

## Data Availability

No new data were created or analyzed in this study.

## Abbreviations

APCs	Antigen-presenting cells
CSF1R	Colony-stimulating factor 1 receptor
CTL	Cytotoxic T lymphocyte
DCs	Dendritic cells
DSS	Dextran sulfate sodium
DSPE	1,2-Distearoyl-sn-glycero-3-phosphoethanolamine
EVs	Extracellular vesicles
IBD	Inflammatory bowel disease
ICAM-1	Intercellular adhesion molecule-1
IFN	Interferon
IL	Interleukin
IL-4Rα	Interleukin-4 receptor alpha
LPS	Lipopolysaccharide
MHC	Major histocompatibility complex
MMP-9	Matrix metalloproteinase-9
MS	Multiple sclerosis
NETs	Neutrophil extracellular traps
NF-κB	Nuclear factor kappa B
OA	Osteoarthritis
pDCs	Plasmacytoid dendritic cells
PEG	Polyethylene glycol
RA	Rheumatoid arthritis
SPAAC	Strain-promoted azide–alkyne cycloaddition
TGF	Transforming growth factor
TEER	Transepithelial electrical resistance
TLR4	Toll-like receptor 4
TNF	Tumor necrosis factor
Tregs	Regulatory T cells
